# Structural bases for neurophysiological investigations of amygdaloid complex of the brain

**DOI:** 10.1038/srep17052

**Published:** 2015-11-26

**Authors:** Liliya B. Kalimullina, Kh. A. Kalkamanov, Azat V. Akhmadeev, Vadim P. Zakharov, Ildus F. Sharafullin

**Affiliations:** 1 Bashkir State University, Department of Physiology, Ufa, 450076, Russia

## Abstract

Amygdala (Am) as a part of limbic system of the brain defines such important functions as adaptive behavior of animals, formation of emotions and memory, regulation of endocrine and visceral functions. We worked out, with the help of mathematic modelling of the pattern recognition theory, principles for organization of neurophysiological and neuromorphological studies of Am nuclei, which take into account the existing heterogeneity of its formations and optimize, to a great extent, the protocol for carrying out of such investigations. The given scheme of studies of Am’s structural-functional organization at its highly-informative sections can be used as a guide for precise placement of electrodes’, cannulae’s and microsensors into particular Am nucleus in the brain with the registration not only the nucleus itself, but also its extensions. This information is also important for defining the number of slices covering specific Am nuclei which must be investigated to reveal the physiological role of a particular part of amygdaloid complex.

Significant interest in understanding the neurophysiology of Am of brain began in the 50^ies^ following development of McLine’s concept of limbic system^1^. It is difficult to overestimate the significance of this concept for the progress in our understanding of brain functions. While initial studies were based on quite simplified view on the organization of Am, a key part of limbic system, accumulated knowledge, that is some case controversial, suggest a more complex picture. Thus, it has become evident that the differentiation of Am in the limbic system’s structure just into two major parts, corticomedial and basolateral, is insufficient. Convincing evidence of a more intricate functional heterogeneity with complex mosaic of groups of neurons differing in phylogenic origin, functionality and information processing organization within the abovementioned parts of Am were presented by many authors including our group in the process of experimental investigations of anatomical specialization of Am^2^,^3^,^4^,^5^,^6^,^7^,^8^.

Given the high specialization and number of different nuclei in the Am, a more detailed neuroanatomical analysis of the results of Am’s neurophysiological investigations is needed. Numerous studies employing various histological and other experimental approaches such as electrophysiological electrodes^6^,^9^,^10^, microdialysis cannulae’s^11^,^12^,^13^, optogenetic fibers^14^,^15^,^16^ and voltammetric microsensors^17^,^18^,^19^ to assess to role of specific nuclei in behavioral or physiological paradigms in awake animals are being performed. Thus, it is critical not only to perform accurate stereotaxic placement of electrodes and microsensors into specific brain area but also to provide the morphological control post-experimentally to verify in which structures of Am (or its part) manipulations were targeted. To achieve both these goals we propose to use highly informative sections of Am to provide mathematically determined objective guideline how to select the most appropriate ones for characterization of a particular amygdaloid nucleus among brain’s series of sections. In essence, this problem is the question of optimization of investigation such as the search of minimal in quantity of section in their totality that provides maximal information about the system studied. As regard to the investigations of Am, we applied the pattern recognition theory^20^,^21^,^22^,^23^, that has not yet been applied for neuromorphological studies. The advantages of this approach lie in the fact that it provides the possibility to pass from the intuitive to the strictly scientifically—based methods of an experimental planning, that is particularly critical for such complex structure as Am.

## Materials and Methods

The investigations were carried out in white rats of Wistar strain 6 month old (total quantity 14, 7 male and 7 female), were obtained from our breeding colony maintained in the Physiology Department of Bashkir State University since 1995. Animals were housed in standard rat cages (3–4 per cage) at 22 °C with continuous access to food and water. Lights were on at 0700 and off at 1900 hours. Animal care and treatments were performed in accordance with the European Convention for the Protection of Vertebrate Animals Used for Experimental and Other Scientific Purposes (ETS N123, Strasbourg, 18.III.1986). All experimental protocols were approved by the Bashkir State University Animal Care and Use Committee.

On the day of sacrifice, animals were injected intraperitoneally (IP) with an overdose of sodium pentobarbital (210 mg/kg). Deep anesthesia was noted by lack of reflexes to tail and foot pinch as well as lack of a corneal reflex. Animals were then perfused transcardially with 0.9% saline (pH 7.4), followed by 10% neutral buffered formalin (pH 7.4; ~150 mL/animal) for 20 minutes. Brains were removed and placed into the same fixative solution 1 month.

After 1 month fixation, brains embedded in paraffin. Brains were then sectioned on a microtome and the continuous series were made of frontal sections (20 μm), which were fixed on covered protein glasses. The slices were deparaffinization, dehydrated in ethanol, placed in water and stained with cresyl violet for Nissl substance, dehydrated in ethanol, cleared in xylene, concluded under the covering glass with Canada balsam.

So, we have obtained a series of approximately 200 sections. The realization of the proposed method is fulfilled via the following successive stages.

The first stage is the selection and mapping of parts of Am, based on general characterization of structural organization of this brain structure. It is carried out on the basis of the classification of nuclei of Am for Krettek and Price (1997) and our experience on neuroanatomy of this brain area^2^,^4^,^24^. As the main object of the investigation was selected cortico—medial group of Am structures that has higher number of nuclei (total 23) in comparison to basolateral group of Am. Nevertheless, basolateral group of Am structures are represented automatically in these section that were analyzed. Each of these structures was assigned an index (x_1_, x_2_, …, x_23_). The definition of the index signs is given in Table 1.

Second stage is tabulation of code table (Table 2) of brain structures that we investigate. While tabulating one must observe the following rules:

- (a) Code signs are forming the table’s columns, j = 1, 2, …, n (in this case x_1_ − x_2_);
- (b) Lines reflect qualitative characteristics of selections and a corresponding set of signs in them;
- (c) The presence of a sign in sections is designated as 1 at the intersection of j-column and i-row, and its absence—as 0;
- (d) The table is based on the study of a continuous series of sections, turned out to be extremely huge, therefore all identical rows are taken out of the table, leaving in it only those which differ, total 14 sections (it is already done in Table 2). The set of these 14 sections is an incompressible block of information.

The observation of these rules guarantees the creation of an incompressible block of information that reflects the essence of structural organization of an investigated formation.

Third stage is calculation of informative weights (P_(j)_) of signs j, j = 1, 2, …, n. Algorithm of estimations’ calculation is used with the use of voting theory^21^,^22^. We give the main formula 1:


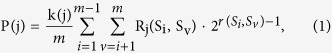


where R_*j*_ (S_*i*_,S_*v*_) = 1 if in the rows S_*i*_, S_*v*_ the meanings of j-columns coincide; R_*j*_(S_*i*_, S_*v*_) = 0—in the opposite case; k(*j*)—is the frequency of meeting of j-sign in a given extract of sections, m—is total number of sections, S_*i*_—is section with the number I; S_*v*_—is section with the number v; *r(S*_*i*_*, S*_*v*_)—is Hemming distance^25^ between sections S_*i*_ and S_*v*_; v—is number of section.

Fourth stage is calculation of sections’ informative weight in made according to the formula 2:


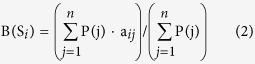


where B(S_*i*_)—is S_*i*_-section’s informative weight, P(j)—is j-sign informative weight, a_*ij*_—is value of j-sign at j-section.

Fifth stage is sections’ distribution according to their informative weights and their distribution according to the informativeness.

Sixth stage is the analysis of the mapping of all essentially important signs in the composition of the chosen highly-informative sections.

## Results

The containing sense of the code signs is given in Table 1.

Table 2 is code table of Am’s structure.

The following meanings of informative weights of P(j)-signs were obtained: P_(1)_ = 765.36; P_(2)_ = 790.93; P_(3)_ = 1055.14; P_(4)_ = 331.64; P_(5)_ = 941.29; P_(6)_ = 790.929; P_(7)_ = 650.79; P_(8)_ = 726.36; P_(9)_ = 286.29; P_(10)_ = 1250.79; P_(11)_ = 1086.79; P_(12)_ = 270.64; P_(13)_ = 968.50; P_(14)_ = 1086.79; P_(15)_ = 1086.79; P_(16)_ = 943.21; P_(17)_ = 410.50; P_(18)_ = 943.21; P_(19)_ = 1157.14; P_(20)_ = 410.50; P_(21)_ = 374.50; P_(22)_ = 1114.00; P_(23)_ = 545.07.

Further, as a result of these calculations the following values were obtained: B_(1)_ = 0.104194; B_(2)_ = 0.191894; B_(3)_ = 0.266264; B_(4)_ = 0.207766; B_(5)_ = 0.257548; B_(6)_ = 0.205362; B_(7)_ = 0.180097; B_(8)_ = 0.251184; B_(9)_ = 0.332378; B_(10)_ = 0.247632; B_(11)_ = 0.207199; B_(12)_ = 0.143046; B_(13)_ = 0.0812847; B_(14)_ = 0.0207627.

While regulating the sections according to their informative balance and distributing them according to their informativeness it was found that they should be arranged as follows: Section № 9 has the informative weight 0.332378; № 3–0.266264; № 5–257548; № 8–0.251184; № 10–0.247632; № 4–0.207766; № 11–0.207199; and so on (see above). The informative weight value gives the possibility to distribute the sections into three groups: 1^st^ (5 sections) with the informative weight more than 0.24; the 2^nd^ (6 sections)—from 0.1 to 0.2; the 3^d^ (3 sections)—lower than 0.1. The first group is highly informative, the second group has middle level informativeness, the third group is low level informative.

The analysis of the representation of all essentially important signs in the composition of the chosen highly-informative sections shows, that only 5 out of the Am series sections (the 3^d^, the 5^th^, the 8^th^, the 9^th^, and the 10^th^) give the information about all essentially important nuclear and screen structures of this formation of brain. So, while investigating structural-functional organization of cortico-medial group of Am structures it is enough to work at the abovementioned 5, but not at 14 sections.

**Figure 1:**
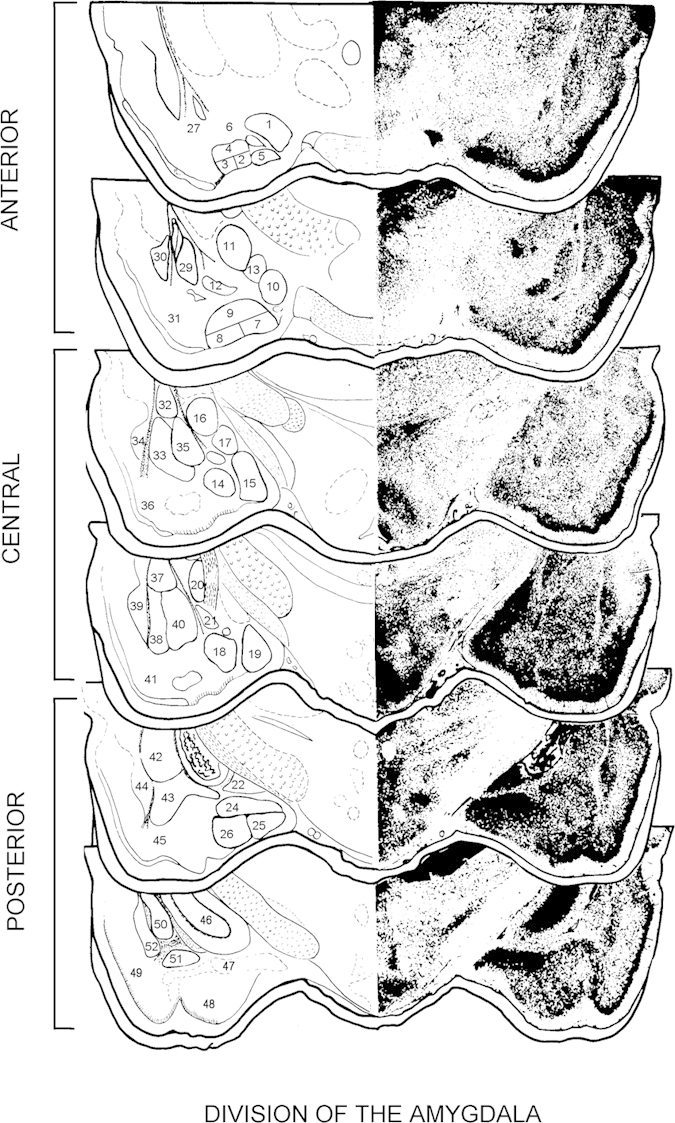
The highly-informative sections of rat’s amygdala (on the basis of pattern recognition theory). Please see Tables 1, 2, 3, 4, 5.

Microphoto of highly-informative Am sections are given in Fig. 1 (list of areas given in Tables 3,4). The use of highly-informative sections of this Am series while fulfilling morphological control of neurophysiological investigations gives the possibility to easily define the topography of a particular nucleus influence on Am even without thorough knowledge of its structure. Furthermore, by determining volume specialization of different nuclei of Am, these data provide guideline for accurate placement of electrodes and sensors into particular parts of Am.

Besides five highly-informative sections that are presented in Fig. 1, we used the sixth section for the representation of the parts which are transitional from Am to hippocampal formation (it is given at the bottom of Fig. 1).

## Discussion

While studying structural-functional organizations of different brain’s areas a morphologist has to work at series of sections. Quantity of these sections is determined by the size of an investigated object, but even in a brain of rather small laboratorial animals (mouse, rat) on condition that selective series are made (if we take every 5^th^, 10^th^, t^h^ section), the number of them will often be more than several dozens. To make an investigation at such number of sections is practically impossible and non-expedient; that is why, before beginning the work, one must select which sections out of the series representing a certain brain are should be investigated to provide most complete information about this brain area characteristics. Incomplete consideration of complex peculiarities of the structural organization of brain areas in physiological investigations often cause the appearance of contradictory facts fruitless discussions and controversial conclusions as evidenced in the literature. This is particularly important for such heterogeneous structure as Am, that cannot be considered as functionally and anatomically unified brain area^5^ due to philogenically determined differences in network organization of various groups of neurons^26^.

The mapping of Am’s nuclei via highly-informative sections is very important not only of histochemical and morphological studies but also for neurophysiological investigations. When carrying out the latter, the proposed scheme of sections of Am can be used:

1. For an exact registration of place of electrodes’ implantation into brain (with the registration not only the nucleus itself but also its parts) by using Paxinos and Watson (1998)^27^ coordinates provided in the Table 5. It should be noted, however, that we present here a more detailed mapping of AM nuclei, including well described Periamygdaloid Cortex^28^, that is not represented in the Paxinos and Watson (1998) atlas.
2. For the defining of the optimal number of sections which must be used for revealing of physiological differentiation, which is present in each concrete structure of Am. So, for example, while investigating the central nucleus, the experiments’ planning must take into account the presence of the rostral part, situated in the anterior part of Am, central part which is on the rostral level of the central part of Am, and caudal, etc.

The authors hope that the proposed model based on of easily reproduced and highly-informative sections of Am can greatly facilitate standardization of Am’s areas that are investigated by physiologist and give the possibility to strictly connect the physiological information obtained to certain structures of this complex. We do hope that the proposed paradigm of Am’s investigations with the use of highly-informative sections will strongly facilitate standartized neurobiological investigations of Am.

While this empirical method applied in this study to map Am nuclei in adult rats, similar approaches can be potentially developed for other mammalian species such as mice, monkeys or even humans in adulthood or at different developmental stages.

The suggested approach is aimed at optimization of experimental protocols focused on understanding the Am functions during physiological or pathological processes. Depending on the technique used one might investigate single Am nucleus or complex set of Am nuclei simultaneously. Mapping specifics of Am nuclei with all its complexity at the level of philogenic origin and functions is particularly important for connectome and neurocomputational studies of neuronal networks.

The work was financially supported by the base part of the Assignments of the Ministry of education and science of the Russian Federation, subject No. 1442.

## Additional Information

**How to cite this article**: Kalimullina, L. B. *et al.* Structural bases for neurophysiological investigations of amygdaloid complex of the brain. *Sci. Rep.* **5**, 17052; doi: 10.1038/srep17052 (2015).

## Figures and Tables

**Table 1:** Coding Am’s structural organization.

Am structures	Code	Am structures	Code
Anterior amygdaloid area	X_1_	Periamygdaloid cortex	X_12_
Nucleus of lateral olfactory tract (cell-dense part)	X_2_	Basomedial nucleus	X_13_
Nucleus of lateral olfactory tract (dorsal part, “cap”)	X_3_	Main trunk of stria terminalis	X_14_
Medial nucleus (rostral 2/3)	X_4_	Intercalated masses (posterior)	X_15_
Nucleus of accessory olfactory bulb	X_5_	Dorsomedial nucleus	X_16_
Anterior cortical nucleus (rostral part)	X_6_	Lateral ventricle	X_17_
Anterior cortical nucleus (caudal part)	X_7_	Posterior medial nucleus (rostral part)	X_18_
Incalated masses (anterior)	X_8_	Posterior medial nucleus (caudal part)	X_19_
Central nucleus (rostral part)	X_9_	Posterior cortical nucleus (medial part)	X_20_
Central nucleus (central/intermediate part)	X_10_	Posterior cortical nucleus (lateral part)	X_21_
Central nucleus (posterior part)	X_11_	Piriform cortex	X_22_
		Bed’s nucleus of stria terminalis	X_23_

**Table 2:** Main code table of structural organization of cortico-medial group of Am.

Sign code	Section number													
	1	2	3	4	5	6	7	8	9	10	11	12	13	14
X_1_	1	1	1	1	0	0	0	0	0	0	0	0	0	0
X_2_	0	1	1	1	0	0	0	0	0	0	0	0	0	0
X_3_	0	0	1	0	0	0	0	0	0	0	0	0	0	0
X_4_	0	0	0	0	1	1	1	1	1	0	0	0	0	0
X_5_	0	0	0	0	1	0	0	0	0	0	0	0	0	0
X_6_	0	1	1	1	0	0	0	0	0	0	0	0	0	0
X_7_	0	0	0	0	1	1	1	0	0	0	0	0	0	0
X_8_	0	0	0	0	1	1	0	0	0	0	0	0	0	0
X_9_	0	0	1	1	1	1	1	0	0	0	0	0	0	0
X_10_	0	0	0	0	0	0	0	1	0	0	0	0	0	0
X_11_	0	0	0	0	0	0	0	0	1	0	0	0	0	0
X_12_	0	0	0	0	0	0	1	1	1	1	1	1	1	0
X_13_	0	0	0	0	0	0	0	1	1	0	0	0	0	0
X_14_	0	0	0	0	0	0	0	0	1	0	0	0	0	0
X_15_	0	0	0	0	0	0	0	0	1	0	0	0	0	0
X_16_	0	0	0	0	0	0	0	0	0	1	0	0	0	0
X_17_	0	0	0	0	0	0	0	0	0	1	1	1	1	0
X_18_	0	0	0	0	0	0	0	0	0	1	0	0	0	0
X_19_	0	0	0	0	0	0	0	0	0	0	1	0	0	0
X_20_	0	0	0	0	0	0	0	0	0	1	1	1	1	0
X_21_	0	0	0	0	0	0	0	0	0	1	1	1	1	1
X_22_	1	1	1	1	1	1	1	1	1	1	1	1	0	0
X_23_	0	0	0	0	1	1	1	1	0	0	0	0	0	0

**Table 3:**
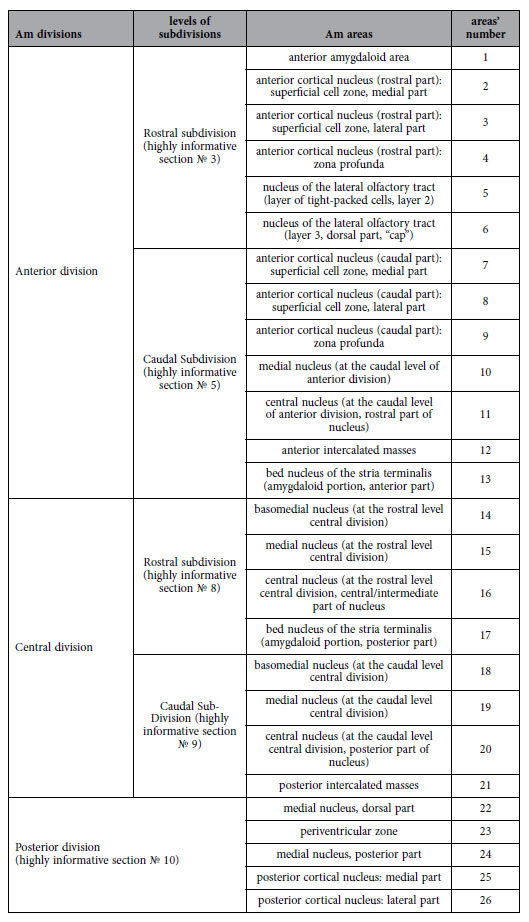
List of the amygdaloid corticomedical areas tabled according to location within the Am divisions and their subdivisions.

**Table 4:**
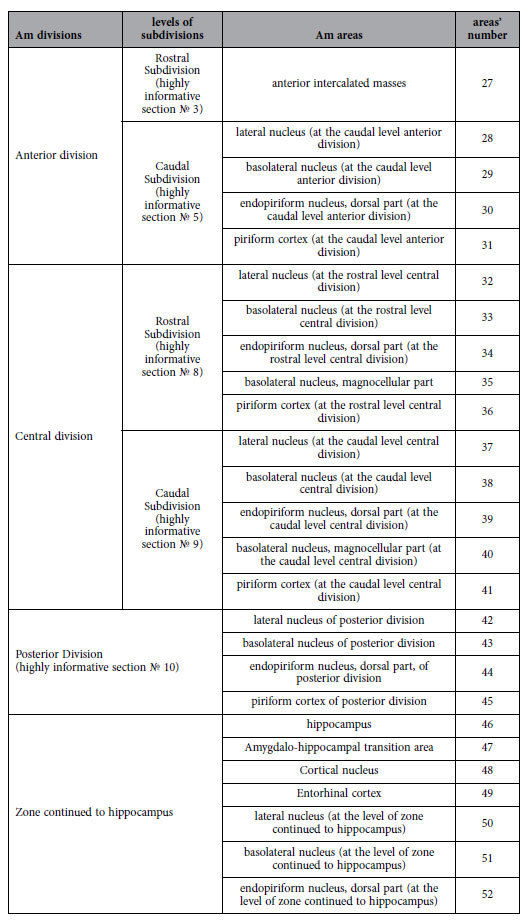
List of the amygdaloid basolateral areas tabled according to location within the Am divisions and their subdivisions.

**Table 5:** Relationship of highly informative sections of rat’s amygdala with the cross-sections of the rat brain atlas from Paxinos and Watson (1998).

Sections of rat’s amygdala in Fig. 1	The figure number and the coordinates relative to bregma in the atlas
Rostral level of the anterior division (highly informative section № 3)	Fig. 23, Bregma –1,30 mm
Caudal level of the anterior division (highly informative section № 5)	Fig. 28, Bregma –2,12 mm
Rostral level of the central division (highly informative section № 8)	Fig. 30, Bregma –2,56 mm
Caudal level of the central division(highly informative section № 9)	Fig. 32, Bregma –3,14 mm
Rostral level of the posterior division(highly informative section № 10)	Fig. 34, Bregma –3,60 mm
Zone continued to hippocampus	Fig. 36, Bregma –4,16 mm

## References

[b1] McLean P. D. The limbic system: Visceral brain and emotional behavior. Arch.Neurol. Psychiat. 73, 130–134 (1955). 13227663 10.1001/archneurpsyc.1955.02330080008004

[b2] Krettek J. E. & Price J. L. Projections from amygdaloid complex to the cerebral cortex and thalamus in the rat and cat. J. Соmр. Neurol. 172, 687–722 (1977). 10.1002/cne.901720408 838895

[b3] Chepurnov S. A. & Chepurnova N. E. Amygdaloid brain complex. Moscow, MGU (1981).

[b4] Akmayev I. G. & Kalimullina L. B. Amygdaloid brain complex: its functional morphology and neuroendocrinology. Moscow, Nauka (1993).

[b5] Swanson L. & Petrovich G. What is the amygdala? Trends Neurosci. 21, 323–331 (1998). 9720596 10.1016/s0166-2236(98)01265-x

[b6] Lyubashina O. & Panteleev S. Effects of cervical vagus nerve stimulatin on amygdala-evoked responses of the medial prefrontal cortex neurons in rat. Neurosci Res. 65, 122–125 (2009). 19523995 10.1016/j.neures.2009.06.002

[b7] Olmos de J. S., Beltramino C. A. & Alheid G. Amygdala and extended amygdala of the rat: a cytoarchitectonical, fibroarchitectonical, and chemoarchitectonical survey. In The Rat Nervous System [ed. Paxinos G. ] [509–603] San Diego, Elsevier Academic Press (2004).

[b8] Sah P., Faber E. S. L., Lopez de Armentia M. & Power J. The Amygdaloid Complex. Anatomy and Physiology. Physiol Re. 83, 803–834 (2003). 10.1152/physrev.00002.2003 12843409

[b9] Bikbaev A. F., Karpova A. V., Chepurnov S. A., Chepurnova N. E. & Kalimulina L. B. Limbic Epileptogenesis: A Model Study Using Kindling from the Amygloid Cortical Nucleus. Dokl. Biol. Sci. 383, 99–102 (2002). 12053580 10.1023/a:1015369319515

[b10] Petrulis A. Neural mechanisms of individual and sexual recognition in Syrian hamsters (Mesocricetus auratus). Behav Brain Res. 200, 260–267 (2009). 19014975 10.1016/j.bbr.2008.10.027 PMC2668739

[b11] Mo B., Feng N., Renner K. & Forster G. Restraint stress increases serotonin release in the central nucleus of the amygdala via activation of corticotrophin-releasing factor receptors. Brain Res Bull. 76, 493–498 (2008). 18534257 10.1016/j.brainresbull.2008.02.011 PMC2474795

[b12] Ebner K., Rjabokon A., Pape H.-G. & Nicolas Singewald N. Increased *in vivo* release of neuropeptide S in the amygdala of freely moving rats after local depolarization and emotional stress. Amino Acids. 41, 991–996 (2011). 21861171 10.1007/s00726-011-1058-0 PMC3172411

[b13] Medina G., Gregoire S. & Neugebauer V. Nasal application of neuropeptide S inhibits arthritis pain-related behaviors through an action in the amygdala. Mol Pain. 10, 32 (2014). 24884567 10.1186/1744-8069-10-32 PMC4046088

[b14] Luna V. M. & Morozov A. Input-specific excitation of olfactory cortex microcircuits. Front Neural Circuits. 6, 69 (2012). 23049500 10.3389/fncir.2012.00069 PMC3446699

[b15] Sparta D. R. *et al.* Inhibition of projections from the basolateral amygdala to the entorhinal cortex disrupts the acquisition of contextual fear. Front Behav Neurosci. 8, 129 (2014). 24834031 10.3389/fnbeh.2014.00129 PMC4018552

[b16] Felix-Ortiz A. C. & Tye K. M. Amygdala inputs to the ventral hippocampus bidirectionally modulate social behavior. J Neurosci. 34, 586–595 (2014). 24403157 10.1523/JNEUROSCI.4257-13.2014 PMC3870937

[b17] Crespi F. Anxiolytics antagonize yohimbine-induced central naradrenergic activity: a concomitant *in vivo* voltammetry-electrophysiology model of anxiety. J Neurosci Methods. 180, 97–105 (2009). 19427535 10.1016/j.jneumeth.2009.03.007

[b18] Crespi F. S. K. channel blocker apamin attenuates the effect of SSRI fluoxetine upon cell firing in dorsal raphe nucleus: a concomitant electrophysiological and electrochemical *in vivo* study reveals implications for modulating extracellular 5-HT. Brain Res. 1334, 1–11 (2010). 20353762 10.1016/j.brainres.2010.03.081

[b19] Barkus C. *et al.* Variation in serotonin transporter expression modulates fear evoked hemodynamic responses and theta-frequency neuronal oscillations in the amygdala. Biol. Psychiatry. 75, 901–908 (2014). 24120093 10.1016/j.biopsych.2013.09.003 PMC4032572

[b20] Dmitriev A. N., Zhuravlev Y. I. & Krendelev F. P. On mathematical principles of classification of things and phenomena. In: Discrete analysis. 7, Novosibirsk, Novosibirsk Mathematical Institut Press, 3–11 (1966).

[b21] Zhuravlev Y. I., Kamilov M. M. & Tuljagonov Sh. E. Formulas for computing measures of feature importance. In: Problems of Cybernetics, Tashkent. ed. IR VTS UzSSR. 44, 15–20 (1971)

[b22] Bushmanov O. N., Dyukova E. V., Zhuravlev Y. I., Kochetkov D. V. & Ryazanov V. V. System analysis and pattern recognition, detection, classification, prediction: Matt. and methods of their use. Moscow, Nauka, 2, 250–273 (1988).

[b23] Zhuravlev Y. I., Ryazanov V. V. & Senko O. V. Recognition. Mathematical methods. Software system. Practical applications. M.: FAZIS. (2006).

[b24] Kalimullina L. B., Akhmadeev A. V., Minibaeva Z. R. & Mutalova L. P. Structural organization of Amygdalod brain complex of the rat. Ross Fiziol Zh Im I. M. Sechenova. 89, 8–14 (2003). 12669587

[b25] Kalkamanov J. A. Numerical methods for constructing correct algorithm of minimum complexity. In: Applied Mathematics. M.: MSPI. 83–91 (1986).

[b26] Kalimullina L. B. What is the cerebral amygdaloid complex. Arkh Anat Gistol Embriol. 99, 85–89 (1990). 2090051

[b27] Paxinos G. & Watson C. The Rat Brain in Stereotaxic Coordinates. 4nd Edn Amsterdam, Academic Press, (1998).

[b28] Scalia F. & Winans S. S. The differential projections of the olfactory bulb and accessory olfactory bulb in mammals. J Comp Neurol. 161, 31–55 (1975). 1133226 10.1002/cne.901610105

